# Vulnerable in silence: Paediatric health in the Ukrainian crisis

**DOI:** 10.1016/j.amsu.2022.104369

**Published:** 2022-08-17

**Authors:** Wireko Andrew Awuah, Jyi Cheng Ng, Aashna Mehta, Rohan Yarlagadda, Kai Sheng Khor, Toufik Abdul-Rahman, Aysha Hussain, Mrinmoy Kundu, Meghdeep Sen, Mohammad Mehedi Hasan

**Affiliations:** Sumy State University, Sumy, Ukraine, Sumy, Ukraine; Faculty of Medicine and Health Sciences, University of Putra Malaysia, Serdang, Malaysia; University of Debrecen-Faculty of Medicine, Debrecen, 4032, Hungary; Rowan University School of Osteopathic Medicine, Stratford, NJ, USA; Faculty of Medicine and Health Sciences, University of Putra Malaysia, Serdang, Malaysia; Sumy State University, Sumy, Ukraine, Sumy, Ukraine; Ross University School of Medicine, USA; Institute of Medical Sciences and SUM Hospital, Bhubaneswar, India; American University of Antigua, St John's, Antigua and Barbuda; Department of Biochemistry and Molecular Biology, Faculty of Life Science, Mawlana Bhashani Science and Technology University, Tangail, Bangladesh

**Keywords:** Armed conflicts, Child health, Paediatrics, Public health, Refugees, Ukraine

## Abstract

The Russian invasion of Ukraine is a humanitarian disaster. It has a wide-ranging impact on the livelihood and the health of those affected by the war. In the midst of constant shelling and casualties, children are more vulnerable to injuries, infections, malnutrition, and trauma, all of which can have serious consequences for their physical and mental health. Children, unlike adults, are simply subjected to the horrors of war with no pre-existing ability to deal with the consequences. We hope to highlight the effects of the current Ukrainian war on the health of the paediatric population, with a particular emphasis on surgical care, cancer care, infectious disease, to name a few. We hope to help contextualize future data and encourage the development of a system to protect and serve the war's most vulnerable population.

## 1 Introduction

The Russian invasion of Ukraine has been a humanitarian crisis on multiple fronts, with many regions of Ukraine ruined because of the war. Among the remnants is the Ukrainian healthcare system-overwhelmed by the constant influx of injuries and deaths. Concerns regarding the Ukrainian health system predate the current conflict– a system undermined by mismanagement, understaffing and underfunding [1]. A program intended to address these concerns was launched in 2014 but has since struggled to come to fruition [2]. The armed conflict has only exacerbated the weaknesses in the health system while impeding efforts to improve it. As of July 25, 2022, the Office of the High Commissioner for Human Rights (OHCHR) recorded an official number of 12,272 civilian casualties, including 5237 deaths [3]. The true statistics is likely to be significantly higher. While there are appropriate concerns regarding the war casualties, one must question the availability of sufficient and able institutions intended to nurse them back to health.

A population demanding our attention is the wellbeing and safety of children in these impacted regions. Children, unlike the adult population, are merely subjected to the horrors of war without any pre-existing ability to handle the consequences due to a lack of independence, experience, and skills needed to navigate a war-torn reality. Previous studies have delineated some of the impacts of war on children's wellbeing in other regions of the world. One study on Sierra Leone showed 47% of their paediatric sample exceeding the threshold for anxiety/depression, 28% exceeded the likely PTSD threshold along with reduced levels of prosocial behaviour [4]. A paper by Medicine Sans Frontiers showed infections were the leading cause of morbidity and exacerbations of chronic conditions such as thalassemia, in analysing children's health during war in northern Syria [5]. Another paper explained that those born during World War 2 had the most negative outcomes, with increased risk of illness and poor functional health [6]. They mention these cohorts were forced to be brought up in a state of damaged processes and broken institutions, paving the way for stunted socioeconomic growth and worsening mental health.

As identified in the reports, among others, armed conflicts pose significant detriment to the growth and development of these children, which can shape their future health and wellbeing. We aim to highlight the impact of the current Ukrainian war on the children of the region. We hope to be able to help contextualize future data and provide a pathway to begin preparing a system that can protect and serve the war's most vulnerable population.

### 1.1 The war's impacts on maternal and newborn health

The United Nations Population Fund (UNPFA) reported that access to maternal care was disrupted for an estimated 265,000 women who were pregnant when conflict erupted [7]. This is supported by the fact that many medical facilities have been hastily relocated into underground metro stations and bomb shelters as health facilities become inaccessible or too damaged to function [7]. Many children are born in bomb shelters, where there is a lack of a reliable power supply [8]. According to the WHO external situation report, unsanitary conditions while delivering newborns underground, due to attacks on hospitals and healthcare facilities, will increase the risk of maternal and newborn complications, illness, and death [9]. UNICEF reported that medical staff are working under intense pressure, and that due to a labor shortage, many are performing multiple roles in the underground suboptimal medical centre [8]. Recent reports have noted the likelihood of preterm births. Preterm babies require specialist care, which is likely to be unavailable during such critical times [9].

The WHO external situation report stated suboptimal vaccination coverage for routine and childhood immunisations, including measles and poliomyelitis [9]. This could lead to outbreaks of vaccine-preventable diseases, and an increase in preventable neonatal mortality. The United Nations Office for the Coordination of Humanitarian Affairs (OCHA) estimates that six million people have limited or no access to safe water, and lack of access to water, sanitation, and hygiene (WASH). This increases the likelihood of foodborne and waterborne diseases, putting vulnerable populations, such as newborns and pregnant mothers, at increased risk of severe illness and death [9]. Unsanitary conditions in healthcare facilities increases the risk of infection in both pregnant women and neonates, leading to life-threatening conditions such as sepsis. Say et al. demonstrated that sepsis is contributory to maternal and neonatal mortality, accounting for 15% of all neonatal deaths and 1 in every 10 maternal deaths [10]. Given all of this, childbirth may be a life-threatening rather than a life-changing experience for some.

### 1.2 Infectious disease outbreaks and respiratory illness

HIV/AIDS rates are expected to rise due to limited access to diagnostic testing and treatment [11]. This could mean that more children will be exposed to HIV through vertical transmission from infected mothers. The risk of waterborne and foodborne disease is increased by a lack of access to water and clean-living conditions. As of May 3, 2022, there had been 115 reported cases of acute watery diarrhoea in children at a refugee centre in Moldova [12]. WHO confirmed the circulation of poliovirus in western Ukraine, which raises concern of international spread in view of the mass migration, subnational gaps in immunization coverage and suboptimal surveillance in neighbouring countries [13]. Even though a vaccine campaign aimed at 140,000 children was launched to increase polio vaccination rates [9], wartime conditions have prevented a successful campaign, and the vaccination rates remain low. A lack of vaccine access and availability means that the most vulnerable members of the population, such as children, are at an increased risk of severe illness and death [9].

The WHO reported that above 98% of Ukrainian children were exposed to levels of fine particulate matter -higher than the WHO air quality guidelines in 2016 [14]. The air quality will be overblown during the Russo-Ukrainian war, with the presence of airborne hazard due to dust emissions from building destruction, and air pollutants generated by fires and explosion of ammunition, worsening the already unsatisfactory situation [15]. The WHO external situation report has revealed several small-scale chemical incidents, including ammonia leaks at industrial sites due to shelling, and there were several fires or explosions at oil or fuel depots reported in areas close to dense population centres [6]. Fires or explosions at fuel depots and destruction of buildings by shelling will create complex mixtures of pollutants that can cause harmful effects to the respiratory tracts [14]. Low levels of air pollution exposure can impede the lung function of children [14]. Poor living conditions such as overcrowding, cold and poor shelter leads to an increased risk of respiratory infections and indirectly leading to acute exacerbation of asthma.

The war's impact on children's livelihoods, and their vulnerability to abuse, resulting in physical and mental injuries, as well as possible post-traumatic stress disorder (PTSD).

Young children exposed to war, conflict, and terrorism are at high risk of developing PTSD, behavioural and emotional symptoms, sleep problems, disrupted play, and psychosomatic symptoms [5,16]. The prevalence of mental disorders (depression, anxiety, PTSD) in conflict-affected children was 22.1% which is significantly higher than the mean global coverage (6.7%) [17]. In a study among former child soldiers and war-exposed civilian children in Nepal, 19% of the total sample reported experience any lifetime suicidal ideation, and a small portion reported suicide attempts [18,19]. A systematic review revealed that about half of children living in conflict experienced PTSD and depression [20]. It is thought that approximately 3.5 million children in Ukraine may develop PTSD and/or depression following the Russo-Ukrainian war [21].

The impact of the Russia-Ukraine war on mental health of children can be due to direct effects from the exposure to traumatic events or violence, or indirectly from displacement, loss of livelihoods, supply shortages and the loss of protective factors such as family and financial stability [22]. The Ukraine war has caused one of the fastest large-scale displacements of children since World War II [23]. The Ukraine war has led to the displacement of 4.3 million children in one month, which already exceeds half of the country's estimated 7.5 million child population [21]. Displaced Ukrainian children are likely to have high levels of anxiety, acute stress, and grief reactions secondary to distress from forced separation from parents [24]. Studies have found that up to half of the children and adolescents refugees resettling in high-income countries have PTSD while up to a third could be affected by either a depression or anxiety disorder or by any other emotional or behavioural problem [20,25]. This poses a mental health burden on the hosting countries.

### 1.3 The current and potential impact of the war on surgical and cancer care

Surgery is a critical component of healthcare that has been shown to suffer during conflict. Contributory factors include limited mobility, destruction and breakdown of health infrastructure, a lack of medical supplies, and a lack of human resources [26]. Hospitals and clinics have shifted their focus to war casualties, resulting in the cancellation of elective surgeries [27]. A single-centre study from Syria found a high incidence of penetrating and complex injuries in children during the civil war [28]. The injury characteristics differed from those of paediatric war casualties in Iraq and Afghanistan, where lower extremity injuries were the most common, followed by head trauma [28]. While we await data on injury characteristics and outcomes of paediatric wartime casualties from Ukraine, these findings can help prepare healthcare workers with management of such patients.

Ukraine has one of the highest childhood cancer mortality rates in the world [29,30]. The ongoing crisis has had a significant impact on children undergoing cancer treatment by interfering with their continued access to therapy. The cessation of healthcare services has caused panic and forced migration in search of medical care [31]. Early detection and regular intervention are essential for efficient and effective cancer treatment, but patients in Ukraine face unfortunate inconveniences.

## 2 Possible long-term implications of the war on children care

The impact of the war will not only have immediate consequences in terms of death and injury tolls, but also long-term consequences for Ukraine's health-care system. The outbreak of the Russo-Ukrainian war will be a significant setback to the country's efforts to implement health reforms and achieve universal health coverage. The Russia-Ukraine war would be detrimental to child development, supported by a study on the impact of civil conflict on child health, which discovered a negative effect of exposure to conflict violence in utero and early childhood on the child's height and weight [32].

The effects of war and forced displacement can have long-term psychological consequences. Factors impacting the mental health of children in Ukraine can be divided into three categories: pre-migration, peri-migration, and post-migration. Pre-migration factors include exposure to war, violence, and poverty; peri-migration factors include exposure to traumatic events like sexual abuse and exploitation; and post-migration factors include education, social support, and parental mental health [25,33]. The psychological impact of migration on the refugee population is proportional to the trauma endured [34]. A cross-sectional study of Syrian refugees in Lebanon found that past war trauma and ongoing displacement have a negative impact on refugee mothers' general mental health, increasing the risk of negative parenting behaviour and, therefore, contributing to poorer psychosocial outcomes for children [35].

### 2.1 Worldwide efforts/response to help Children's health in Ukraine

As the conflict between Russia and Ukraine worsens, causing serious and long-term harm to children around the world, several humanitarian organisations have stepped forward to lend a helping hand. UNICEF and partners are working to provide essential services to vulnerable children and families, such as health, education, protection, water, and sanitation, as well as life-saving supplies [36]. Life-saving health and medical supplies had been distributed to reach around 3.8million and families [36]. While the UN continues to distribute water and hygiene items, it is also increasing the number of mobile child protection teams operating within acute conflict zones [37].

UNICEF assisted 35,000 households with multi-purpose cash assistance and have helped over 3.1 million people with safe water access [36]. UNICEF, the UN refugee agency, and the UNHCR have collaborated with governments and civil society organisations to establish “Blue Dots,” or safe zones for children and families along the border [38]. “Blue Dots” will provide the following services: information and advice desks, child-friendly rest and psychosocial support spaces, family reunification services, counselling and psychosocial support for both children and parents/caregivers.

### 2.2 Recommendations

As illustrated by this paper, the paediatric population represents one of, if not the most, vulnerable groups affected by the Ukrainian crisis. There comes a need for guidelines to help protect and maintain the wellbeing of children in the context of armed conflicts and crises.

Ottolini et al. [39] emphasises that the factors most contributing to the high prevalence of infectious disease in war-affected paediatric populations is due to a lack of access to safe shelter, adequate nutrition, hygiene, and healthcare. A multipronged approach targeting each of these factors may address tackling infectious diseases. The most straightforward action might be to offer safe shelter and nutrition in demilitarized/neutral zones. Acute respiratory infections, varicella, and diphtheria are among the most common infections [39]. As several of these are vaccine-preventable, there is a reliance on NGOs, international organisations, and countries to support vaccine provision not only for Ukrainian children, but also to close the vaccine coverage gaps in neighbouring countries to limit transborder transmission secondary to the mass population movement.

Military hospitals in war-torn regions often admit paediatric patients with medical emergencies. A paper studying activity at a German field hospital demonstrated a need for military medical facilities to be adequately prepared and staffed to be able to provide care to paediatric patients [40]. The U.S. military includes paediatricians who may either stay in the US to provide care and support to the child(ren) of military families or may be deployed to care for the servicemen who are often young. We may therefore recommend a similar sentiment for other militaries that do not already provide this care and would recommend that these militaries provide the children of the affected region access to these deployed paediatricians as well.

Lastly, another recommendation could be to utilise telemedicine powered camps to provide paediatric care. Ghbeis et al. [41] describe a preliminary trial of a tele-paediatric intensive care unit to expand the war-torn affected children's access to care. Under the guidance of a telemedicine physician, other personnel may be trained to carry out the actions recommended by the physician.

These suggestions are not exhaustive and represent a few potential ways to maintain paediatric care in the event of disruption by war or armed conflicts. We would suggest further research, discussion, and guideline formation to help address paediatric care in conflict zones.

## 3 Conclusion and outlook

Russia's invasion of Ukraine has been a humanitarian disaster. With a bleak picture of the conflict's aftermath, the paediatric population is the most vulnerable, with a high prevalence of injury, infection, mental health issues, and inadequate nutrition. To ensure their well-being, they require adequate shelter, nutrition, sanitation, and healthcare. Implementing these measures will necessitate a global collaborative effort, which is urgently needed.

## Ethical approval

N/A.

## Sources of funding

N/A.

## Author contribution statement

Wireko Andrew Awuah, Jyi Cheng Ng, Aashna Mehta conceptualized the topic. All authors contributed to reading, writing, editing the original draft and critical revision under the supervision of Mohammad Mehedi Hasan. All authors read and approved the final version of the manuscript.

## Registration of research studies

Name of the registry: N/A.

Unique Identifying number or registration ID: N/A.

Hyperlink to your specific registration (must be publicly accessible and will be checked): N/A.

## Guarantor

All authors.

## Consent

N/A.

## Data availability statement

No data available.

## Conflicts of interest statement

Authors have no conflicts of interest to declare.
